# Managing Sustainable Working Hours within Participatory Working Time Scheduling for Nurses and Assistant Nurses: A Qualitative Interview Study with Managers and Staffing Assistants

**DOI:** 10.1155/2023/8096034

**Published:** 2023-12-09

**Authors:** Majken Epstein, Erebouni Arakelian, Philip Tucker, Anna Dahlgren

**Affiliations:** ^1^Department of Clinical Neuroscience, Division of Psychology, Karolinska Institute, Solna 171 65, Sweden; ^2^Department of Surgical Sciences, Uppsala University, Uppsala University Hospital, Uppsala 751 85, Sweden; ^3^School of Psychology, Swansea University, Swansea SA2 8PP, UK; ^4^Stress Research Institute, Department of Psychology, Stockholm University, Stockholm 106 91, Sweden

## Abstract

**Aim:**

To bring insights into how healthcare managers and staffing assistants work to achieve sustainable working hours within a participatory scheduling system.

**Background:**

Hospital nurses and assistant nurses often work on rotating shifts, which affects their opportunities for sleep, recovery, and work-life balance. In Sweden, a participatory scheduling approach is commonly used, where working hours are planned in collaboration between employees, managers, and staffing assistants. Influence over working hours is related to positive outcomes among shift workers. However, it also places responsibility on the employee to schedule working hours that promote health and patient safety, i.e., sustainable working hours. Accordingly, the organisation has responsibilities to support the employee in this regard.

**Methods:**

Semistructured individual interviews were conducted with 11 managers and 9 staffing assistants from four Swedish regions and analysed using thematic analysis.

**Results:**

Several key factors for achieving sustainable working hours within the context of participatory scheduling were described: distribution and clarity of responsibilities, allocating time for scheduling, establishing shared responsibility, considering fairness, fostering an individual relationship with the employee, managing dissatisfaction, providing support, clarifying guidelines for sustainable scheduling, managing inconsistencies between employee requests and sustainable working hours, and considering recovery opportunities and the competence mix on shifts. Additionally, contextual factors, such as staffing levels, working procedures, working time arrangements for night work, and technological support, were highlighted as important.

**Conclusion:**

Achieving sustainable working hours within participatory scheduling involves considering the interactions between factors at the levels of the organisation, the individual, and the technological systems. *Implication for Nursing Management*. Nurse managers and staffing assistants must work closely with their employees during participatory scheduling to ensure sustainable working hours. Key goals in this regard include establishing a shared responsibility, clarifying responsibilities and guidelines for sustainable scheduling, and allocating time for the scheduling process.

## 1. Introduction

Healthcare organisations operate 24/7, which requires shift work. In the European Union, about 40% of healthcare workers are exposed to shift work [1]. Among nurses and assistant nurses, rotating shift work is common, alternating between morning, evening, and night shifts. Working hours affect sleep and recovery [2] and maintenance of a work-life balance [3], which are important factors for employees' health [4] and intention to stay in the organisation [5, 6]. Furthermore, insufficient sleep and recovery cause fatigue which can be a patient safety hazard [7]. Thus, working hour arrangements are an important consideration for healthcare organisations in order to ensure employee health and patient safety, as well as managing the world-wide challenges with recruitment and retention of staff in healthcare [8, 9].

In participatory working time scheduling (hereafter referred to as participatory scheduling), working hours are planned with the aim of meeting both employees' individual preferences and the wards' specific staffing needs. Usually, ward managers, staffing assistants, and employees cooperate in the schedule planning, which often takes place in several steps and in cycles of negotiations and adjustments [10, 11]. Participatory scheduling implementations vary, e.g., regarding how the process is organised, degree of employee influence, and ward-specific scheduling rules. Participatory scheduling is commonly used among shift-working nurses and assistant nurses in Sweden, although there is a heterogeneity in how it is implemented [12].

Influence over working hours has been related to several positive outcomes among shift-working healthcare employees, such as higher job satisfaction [13], improved work-life balance [14], reduced fatigue after work [15, 16], reduced risk of short sleep and poor workability [17], and higher self-rated quality of care [18]. Use of participatory scheduling was also found to be related to decreased sickness absence among nursing staff [19]. Furthermore, satisfaction with schedule flexibility has been related to lower intention to leave the workplace [20], while being forced to work night shifts has been cited as a reason for leaving the workplace [21].

Concerns have been raised that employee influence over scheduling could result in working hours that impair recovery and health, e.g., through prioritisation of social activities over recovery, sleep, and health [22]. While such concerns have been realised in some studies, e.g., an increase of long work shifts [10, 15], other studies have found few such unfavourable effects of participatory scheduling [23]. A similarly mixed picture emerges with regard to the effects of participatory scheduling on job satisfaction [24].

Given some contradictory results regarding the impact of participatory scheduling, it is important to identify which factors are important for the successful implementation of participatory scheduling. Key enablers of implementation, as identified by a recent systematic review [25], were an understanding among the employees that the process will not always run smoothly and changes will become necessary, having a team-based approach involving all employees, continuous support and involvement of the head nurse, assessing the nursing workload before implementation, and using a computerised self-scheduling system. Examples of barriers were when nurses see self-scheduling as an individual entitlement (instead of a joint agreement to enhance both the employee's life and the ward's functioning), organisations underestimating how sensitive the issue of scheduling is for employees, favouritism by the schedulers, and staffing shortage. The review's findings highlighted the importance of the implementation process and contextual issues, for the success of participatory scheduling.

Previous research has indicated that certain shift schedule characteristics are associated with sleep and fatigue problems, such as a high frequency of quick returns (<11 hours between working shifts) [26, 27], many consecutive working days [28], night work [29], and backward rotation of shifts (night-evening-day) [30]. Also, night shifts per se [31], >3 consecutive night shifts [31], quick returns [32], and long working hours [31, 33] have been related to increased risk for occupational injuries. Among nurses, long working hours (>12 h) [34], a high frequency of quick returns [35], and night shifts [36] have also been associated with a higher risk for medical errors, with fatigue as one plausible mechanism [36]. Furthermore, a single day off after night work seems insufficient to fully recuperate with respect to alertness [37] and cognitive function [38].

Support exists for an association between shift work and future development of chronic diseases (e.g., type 2 diabetes, coronary heart disease, and cancer) with higher risks for shift work including night work, with disturbed sleep and circadian disruption as plausible mechanisms [4]. Regarding night work, >9 h shift duration [39], >3 consecutive shifts [39], and <28 h rest after the last night shift [40] have been associated with increased risk for disease development.

Accordingly, schedule design in shift work has health and safety implications for both employees and organisations. In this article, we define sustainable working hours as working hours that promote both short- and long-term employee health, sleep, and recovery, as well as patient safety. The requirements for sustainable working hours, as identified by previous research, are that the shift schedule should limit the number of consecutive shifts [28] and quick returns [26, 27, 32, 35]; limit the length of shifts [31, 33, 34]; limit consecutive night shifts to a maximum of 3 [39]; enable sufficient (>48 hours) rest time after night work [41]; and feature forward rotation of shifts [30].

Leadership behaviours characterised by consideration and support, and a good quality leader-employee relationship, are positively related to employee well-being and lower stress levels [42]. Recent studies have also suggested that leadership is an important factor in facilitating employees' sleep. The concept of sleep leadership has been defined by a set of behaviours in which leaders both encourage and enable employees to obtain healthy sleep [43]. A series of studies among military personnel indicates that employees' experience of sleep leadership is associated with higher subjective sleep quality and sleep quantity [44], as well as with less sleep disturbance and sleep-related impairment during daytime [45].

The current project uses the human-technology-organisation concept as its theoretical basis, to understand the role of the individual in the complex organisation of healthcare. The concept suggests that work activities can be described, analysed, and understood by describing the interactions between the three subsystems—human (also referred to as “individual” in this work), technology, and organisation [46]. These subsystems play a key role in participatory scheduling, where both employees and the organisation are involved in planning of working hours, often using computerised scheduling systems.

Given the importance of working hours for both employee health and patient safety, and the widespread use of participatory scheduling among healthcare personnel, there is a need to understand how to optimise participatory scheduling models while ensuring sustainable working hours. The aim of this study was to bring insights into how healthcare managers and staffing assistants work to achieve sustainable working hours within a participatory scheduling system.

## 2. Materials and Methods

### 2.1. Design

This qualitative descriptive study examined participants' experiences and thoughts [47], as part of a larger project investigating how healthcare organisations can achieve sustainable working hours. The study adhered to the Consolidated criteria for reporting qualitative research (COREQ) [48], see Appendix 2.

### 2.2. Context

Four regions in Sweden (including one metropolitan region) were represented in this study. There were a variety of ways of organising the scheduling process, involving the manager, the employees, one or more staffing assistants, and/or scheduling groups (a group of nurses or assistant nurses who had time designated for work with scheduling). Schedules were planned for between 5 and 12 weeks at a time (up to 16 weeks during summertime). A scheduling period commonly started with a *planning period*, where the employees proposed which shifts they would like to work during the coming period, either using paper and pen, a whiteboard, or a technological system (“Tessa,” “Heroma” and/or “Adacta”). There were rules about a minimum number of certain shifts (evening, weekend, and/or night shifts) in each scheduling period which employees had to follow. Also, the employees were allowed to place “vetoes” on shifts they did not want to work (varying between 1 veto/week and 3 vetoes per 10 weeks). After the planning period, the *adjustment process* started and lasted for between 1 and 3 weeks, where staffing shortages and excesses on shifts were identified and shift changes were made to fulfil staffing and competence needs on each shift.

The first part of the adjustment process was carried out by the employees themselves. In some wards, a scheduling group or staffing assistant was responsible for either the whole adjustment process or for making further necessary adjustments after the employees' own adjustments. Final approval of the schedule was given by the manager, or in some cases by staffing assistants, although the manager had the formal responsibility for the schedule. Sometimes the planning and adjustment process was divided into two steps, where planning and adjustment of weekend and/or night shifts were made in a first step, and the remaining shifts in a second step.

### 2.3. Participants

Purposive sampling was used to obtain participants from diverse regions in Sweden. Inclusion criteria were first-line managers and staffing assistants who worked actively with working time scheduling and used participatory scheduling. Twenty-seven participants were invited, of whom eleven first-line managers and nine staffing assistants accepted. The participants were 19 women and one man, aged between 28 and 61 years (*M* = 46) and had worked with planning work schedules between 3 and 30 years (*M* = 9). Managers' professions were registered nurses (*n* = 7), specialist nurses (*n* = 3), and midwife (*n* = 1). Staffing assistants' professions were assistant nurses (*n* = 7), behavioural scientist (*n* = 1), and unknown (*n* = 1). Education about working hours and scheduling varied, with participants often being introduced by their predecessor who educated them in the scheduling software and informed them about working time regulations. A few (*n* = 6) had received an education about “healthy working hours.” The participants worked at wards with the following medical specialties: neurology, maternity, pulmonary medicine and hematology, orthopedics, medicine, oncology, pediatric emergency, medical emergency, and medical intermediate care.

### 2.4. Data Collection

The participants were contacted by the research group through their electronic work e-mail addresses and informed about the aim of the study. After receiving written informed consent, the last author (associate professor with previous experience of qualitative semistructured interviewing and analysis) and a master's student, trained and supervised by the last author, conducted the interviews, which took place during March 2020–October 2021 using face-to-face (*n* = 4), phone (*n* = 15), and video call (*n* = 1) methods. The participants chose the interview method and location (their homes or workplaces). The interviews which lasted between 24 and 73 minutes (*M* = 47) were audio-recorded and transcribed verbatim for further analysis. The interviews were conducted in Swedish, which was the native language of the informants participating in the study and the interviewers.

### 2.5. The Interview Guide

An interview guide with semistructured open-ended questions was designed for the purpose of this study. The guide started with demographic questions, followed by nine (staffing assistants) or ten (managers) main questions about the work scheduling process, follow-up procedures, rules and regulations, challenges and need for support, ideas for improvement, technical support, and eventual conflicts during scheduling. Probing questions such as “please tell more/explain more” were used to deepen the discussions in the interviews. Participants were asked to focus on work procedures during normal operation and not during the peaks of the COVID-19 pandemic. The questions differed slightly between managers and staffing assistants (see Appendix 1).

### 2.6. Data Analysis

Data were analysed using the six phases in thematic analysis according to Braun and Clarke [49] (see Table 1). Initial coding and searching for themes were conducted in Swedish. From phase 5, defining and naming themes, and during the rest of the process, English language was used. All authors who analysed data were fluent in Swedish and English in both writing and speech. Experiences referring to the working hours and scheduling during the COVID-19 outbreak and peaks were identified and excluded from this analysis. The first author (MSc, licensed psychologist) coded all the interviews. The second and the last authors coded 50% of the interviews each, independently. The second author is an experienced qualitative researcher (associate professor), who confirmed the coding structure and the analysis process. The final themes are a result of several discussions between all authors. The first interview was treated as a test interview, meaning that the participant was asked if he/she understood all questions asked and whether the order of the questions felt relevant. After the first interview, the authors reviewed the interview guide regarding whether answers were received on what was sought by the questions in the guide. As no major changes were made to the interview guide, the first interview was also included in the data analysis. During the last interviews, no new information was identified and the research team considered that data repeated itself in the last interviews.

### 2.7. Ethical Considerations

This study was approved by the Swedish Ethical Review Authority (2019-05245). The study followed the Declaration of Helsinki regulations [50] and local ethical guidelines and regulations [51].

## 3. Results

Four themes and fourteen subthemes were identified (see Table 2). The results described are from both managers and staffing assistants' viewpoints. Differences in their experiences are pointed out with subheadings or in texts.

### 3.1. Organisation of the Scheduling Process

#### 3.1.1. Distributed Responsibilities and Decision Making

Responsibility for the schedule was usually distributed between different persons. Commonly, the staffing assistants and/or scheduling groups did much of the administration, scheduling adjustments, and communication with employees, but it was sometimes undertaken by managers. Participants felt that some employees did not engage sufficiently in the process, e.g., not adjusting the schedule to fulfil staffing needs during specific shifts or not complying with rules during planning. The manager usually had a continuous dialogue with the staffing assistant or scheduling group during the process and was often more directly involved during difficult situations, such as when the staffing assistant and/or scheduling group could not find a scheduling solution or when employees expressed high dissatisfaction.

The views of managers and staffing assistants, respectively, are described below.

*(1) Managers' View*. Some managers perceived the scheduling groups' work as unsatisfactory, such as planning schedules without enough recovery opportunities. Managers reported identifying working hours with a potential risk for health and/or safety, such as many consecutive shifts or insufficient competence mix on shifts, after the employees' and staffing assistant's adjustments. The managers who did not have a formal staffing assistant reported needing support in the scheduling process due to the large number of employees: “*it is impossible for me as a manager to check a group of 70 people*” (Manager 6). Attitudes towards involvement in scheduling varied. Some felt that this was an important part of their leadership, providing insight into the employee's schedule and having positive effects on the employee-manager relationship, which in turn made the scheduling process smoother. Others felt that responsibility for the schedules should be allocated to staffing assistants: “I *don't think managers should work so much with schedules (…) it could actually be done by staffing assistants*” (Manager 8).

*(2) Staffing Assistants' View*. Staffing assistants often described themselves as intermediaries between employees and the manager. This could be challenging as they received opinions and criticism regarding the schedule from employees, yet they had no formal mandates to meet these, or make final decisions. Moreover, sometimes they were a part of the group of employees that were being scheduled which made it difficult to stay neutral. Some experienced good collaboration and support from managers, whereas others did not: “*you have no answer as a staffing assistant (to give the employees) (…) it means that the manager must be engaged and offer support*” (Staffing assistant 3). Often the staffing assistants had the role of asking employees to work extra shifts, which was experienced as emotionally demanding when they knew the employees were tired. In those cases, support from the manager was important.

#### 3.1.2. Time-Consuming

Much time was spent on scheduling, both by managers, staffing assistants, and employees. One manager recounted that ”*when you are done with one (scheduling period), you almost have to start with the next, it takes a lot of time*” (Manager 2). A few managers questioned the benefits of participatory scheduling given how time-consuming the process was. Other managers, and staffing assistants, thought the time spent was worth it for the benefits it brought employees having influence over their working hours. It was also discussed that employees and/or scheduling groups did not have enough time allocated for scheduling, which was suggested as one explanation for why the employees' engagement in the scheduling was sometimes insufficient.

### 3.2. Active Leadership

#### 3.2.1. Establishing a Shared Responsibility Framework and Fairness

The importance of establishing a shared responsibility framework in scheduling, between the workplace and the employees, was emphasised. Some perceived that the employees expected to freely choose their working hours, a misunderstanding that was counteracted through continuous communication about the importance of “*giving and taking*” (Staffing assistant 5). The scheduling process could also be made smoother by pointing out to employees that they had ample possibility to influence their working hours and showing them that the workplace aimed to be highly flexible in meeting employees' requests. Other ways of establishing a shared responsibility framework were to gather the whole staffing group to discuss solutions to scheduling issues, e.g., if many employees had applied for vacation during the same weeks.

Respondents also emphasised the importance of fairly distributing unpopular shifts, typically evening, night, and weekend shifts, and public holidays. For example, if a day shift was overstaffed during the adjustment period, the choice of which employee should be moved to the evening shift the same day might be based on who worked the least evening shifts in that scheduling period. Fairness also played a role in determining how many changes were made in the employees' proposed schedules. Some respondents reported that they kept track of how many changes were made in each individual schedule during each scheduling period and tried to even that out in the coming periods. If changes were needed to fulfil staffing and competence needs on a shift, the process began with the adjustment of schedules of those employees who had not engaged in the scheduling process.

#### 3.2.2. The Individual Relationship with the Employee: Continuous Dialogue, Mutual Problem Solving, and Adaptations

There was an emphasis on the importance of the individual relationship with the employee. This provided insight into individual life circumstances, preferences, and tolerance for shifts and shift combinations, which could be considered in scheduling, for example, making special adaptations in the schedule if the employee had experienced a significant life event, or letting an employee work only day shifts every other week for private reasons. It was considered to be important to have an open dialogue regarding the employees' working hours, as this gave insight into the employees' schedules, workload, and need for recovery. This also facilitated mutual problem solving and discussions about the importance of sustainable scheduling. Participants felt that it was important that the staffing assistants were easily accessible to the employees.

Managers described continuously looking at their employees' schedules and sometimes had to remind them about recommendations for sustainable scheduling. Some managers also reported that they investigated the employee's past and current working hours if the employee seemed to feel unwell, and that they had noted potential associations between compressed working hours and sick leave. Moreover, working hours were discussed during the yearly staff appraisal.

Staffing assistants sometimes had knowledge of individuals' weekly leisure activities and took those into consideration in the planning. However, having a lot of private information about the employees could made the work more difficult: “*it was easier in the beginning when you had no idea, now I know that this person goes riding Monday evenings (…) and he doesn't want to work evening-day, and she doesn't want to work day-evening (…) it is a lot*” (Staffing assistant 4). Moreover, dialogue with employees was described as having positive consequences for sustainable working hours:

“*I have talked to them (…) the schedules are looking much better. They (the schedules) were awful (when I started working here), it was every weekend, and it was many consecutive shifts (…) because nobody had talked to them*.” (Staffing assistant 8)

#### 3.2.3. Managing Dissatisfaction

It was reported that working hours and influence over scheduling were of great importance for many employees and sometimes provoked strong feelings. Dissatisfaction was sometimes expressed by employees when their scheduling requests were not met, and it was described as “*difficult making everyone satisfied with their schedule*” (Staffing assistant 6). An uneven distribution of weekend shifts or unmet scheduling requests could also cause dissatisfaction, and work during public holidays could provoke strong feelings. Dissatisfaction was managed by explaining and giving a rationale for the shift changes. Another approach was to highlight to the employee the extent to which their scheduling requests had been met. In wards where the technological system made much of the adjustment process automatically, problems with employees' experiences of injustice with scheduling had decreased.

#### 3.2.4. Education, Support, and Clear Scheduling Rules

New employees were given an introduction to the scheduling process, including information about rights and obligations and the importance of recovery. Sometimes, all employees were offered continuous support from staffing assistants, and scheduling was a recurrent topic in workplace meetings. Communication about rules (e.g., number of weekend shifts, vetoes, etc.) facilitated the scheduling process. It was communicated to the employees that to be fully guaranteed days off, the employees had to use vacation days instead of vetoes, but also vetoes were commonly approved. More education of the employees about scheduling and implications for health was needed, in order to increase “*understanding of the body and the circadian rhythm (in relation to scheduling*)” (Manager 11).

### 3.3. Balancing Sustainable Working Hours, Employees' Scheduling Requests, and Competence Needs

#### 3.3.1. Official/Unofficial Guidelines for Sustainable Working Hours

Guidelines for sustainable working hours were communicated to the employees and considered during the adjustment process. A majority had guidelines for a limit of weekly working hours and a maximum number of consecutive work shifts (usually five or six). Other guidelines were for a minimum of two consecutive days off, a maximum number of consecutive night shifts (often three), 48–72 hours off after working night shifts, and forward rotating shifts, i.e., day-evening-night. A minority described a lack of guidelines for sustainable working hours. While some workplaces had stricter guidelines, others let the employees choose how to relate to them, i.e., the guidelines were more unofficial:

“*We have presented research about healthy working hours (…) but we give them (the employees) the freedom to schedule as they like (…) we have no rules prohibiting them to plan as they want anyway*.” (Manager 3)

Shift combinations with quick returns (usually an evening shift followed by day shift resulting in <11 hours between shifts) were discussed with varied attitudes and recommendations. Some encouraged employees to try to avoid or minimise quick returns and informed them about potentially negative health effects; others lacked guidelines regarding these. Some emphasised the problem with general guidelines about quick returns, referring to individual variances in tolerance.

#### 3.3.2. Employees' Scheduling Requests versus Sustainable Working Hours

Many participants reported that the employees themselves took responsibility for self-scheduling sustainable working hours. However, examples were given of self-scheduled unsustainable working hours, such as compressing working shifts in order to get longer continuous periods of time off. Several managers and staffing assistants attached importance to the employees' freedom in the scheduling process, stating that potentially unsustainable working hours (e.g., 7–10 consecutive shifts, double shifts, working a day shift the day after leaving the night shift, and quick returns) were accepted if the employee had chosen it. It was stated that “*if they themselves have proposed an unhealthy schedule (…) I do not change it automatically, then you have lost the point of having an individual schedule*” (Manager 3), and that tolerance and what is experienced as a healthy schedule could vary between individuals. However, not all had this approach, and some clearly stated that sustainable working hours had the first priority regardless of the employees' scheduling requests.

#### 3.3.3. Considering Recovery Opportunities

Recovery opportunities were considered important in scheduling. One staffing assistant discussing the adjustment process described having “*a checklist for healthy working hours (…) how many consecutive shifts, how much daily rest and weekly rest*” (Staffing assistant 5). To plan schedules with enough recovery in-between shifts for employees working full-time on rotating three shifts was described as a great challenge by staffing assistants. Overstaffing of weekday shifts was sometimes necessary to facilitate an even distribution of recovery among individual schedules.

#### 3.3.4. Competence Mix on Shifts

Competence mix on shifts was considered during the adjustment process. In some wards, a competence grading based on experience was used, with the aim of covering every shift with a mix of new and more experienced employees. Sometimes, this was difficult due to high staff turnover and that “*new nurses are starting all the time*” (Staffing assistant 2). Sometimes, employees wanted to choose which colleagues to work with, which could result in an insufficient competence mix (e.g., many new employees working the same shift).

### 3.4. Contextual Factors

#### 3.4.1. Staffing Levels, Short-Term Absence, and Solutions

Staffing shortage and high turnover rates were described as major barriers for achieving sustainable working hours. Understaffing led to difficulties meeting employees' shift requests, irregularity in individual schedules, less approved vacations, more overtime work, and shifts with insufficient competence mix. Understaffing on shifts also reduced recovery opportunities for employees during shifts. Covering night and weekend shifts was a big challenge. Furthermore, staffing shortage became a serious problem during short-term absence causing shift vacancies, which were described as “*a permanent stressor*” (Staffing assistant 1), and covering night shift vacancies was especially difficult. At the few wards where the staffing level was described as sufficient, the scheduling process also worked better.

Various attempts were made to manage problems with understaffing and shift vacancies, for example, reducing the number of hospital beds, having part-time employees covering weekend and night shifts, having a local nurse/assistant nurse substitute pool, or hiring temporary agency nurses/assistant nurses. Another strategy involved forecasting workload peaks and planning for higher staffing levels in advance. Some interviewees reported sharing staff with adjacent wards. However, regarding employees rotating to other wards, one manager noted that “*it's a disadvantage to not have a full overview of the employees' working hours (including overtime work)*” (Manager 6).

Specific solutions for short-term shift vacancies included borrowing employees from other wards (however, many employees disliked this), moving employees from upcoming overstaffed shifts, asking employees to work the vacant shift instead of a coming shift (i.e., postponing the vacancy), or asking employees to work extra shifts or to stay and work until the vacancy was filled. Working extra shifts could lead to guidelines for sustainable scheduling being breached. Before asking employees to work extra shifts, individual life circumstances and recovery opportunities in the schedule were considered. At some wards, employees could choose not to be asked to work extra shifts. It was reported that employees usually cooperated and were helpful with covering shift vacancies. One staffing assistant thought that it was “*difficult for the employees to say no, when they know how high the workload is when you are understaffed*” (Staffing assistant 1). Sometimes, there were employees who were willing to work many extra shifts. While there was an ambition not to ask employees who had worked many extra shifts recently, sometimes there was no choice: “*but that is very difficult, because if no one wants to work an extra shift, and the patient safety is threatened, you choose the person that says yes*” (Manager 9). Double shifts were avoided, if possible, but they could occur in periods with high workload and/or many shift vacancies, if the employee agreed.

The scheduling process for temporary agency personnel was sometimes organised differently, as they covered the shifts that the ordinary employees opted out of. They tended to work a lot of overtime, double shifts, and inconvenient working hours. One staffing assistant reported having a poor overview of the temporary personnel's working hours:

“*They (temporary personnel) usually have one or two other workplaces that they go to, it feels like they work all the time. (…) what they do in other places, I don't know if they work 31 days in a row*.” (Staffing assistant 8)

#### 3.4.2. Working Procedure at the Wards

How care was organised influenced the need for quick returns. Continuity of care was facilitated if employees on the morning shift had also worked the evening shift the day before. It was believed that some employees preferred that because “*they want that overview in the morning (…) a lot happens in a short time in the morning, and they have to do all their tasks and be prepared for the round, which starts quite early*” (Manager 11). To reduce the need for quick returns, other managers described changes such as efficient procedures for handing over between shifts, i.e., verbal reporting or bedside reporting, and standardised documentation templates stating what was last done and what needs to be done next, thus facilitating working morning shifts without quick returns.

#### 3.4.3. Working Time Arrangements for Night Work

It was common for employees to get a reduction in their working hours if they worked night shifts. However, it was reported that many employees felt that they had to work a very high number of night shifts to get a satisfying working hour reduction, which some experienced as too burdensome and therefore had left the workplace. In some wards, full-time night workers were hired to cover night shifts. This was experienced as a good solution since working rotating three shifts was seen as strenuous. In some wards, employees who had been identified as having low night shift tolerance were excluded from night work, while other wards shared night shifts among all employees irrespective of tolerance: ”*from a safety perspective, the nights are not perfect, especially when you force people to work night shifts (…) who have been awake for 24 hours when they come to work*” (Manager 6).

#### 3.4.4. Technological Enablers and Barriers for Sustainable Working Hours

Technological systems were widely used in the scheduling process and were experienced as time saving and helpful. They could facilitate the creation of sustainable working hours by automatically generating and adjusting schedules based on predefined settings, such as individual general preferences (e.g., avoidance of certain shifts), employees' shift requests, staffing needs and competence, guidelines for sustainable working hours, and working time regulations. Technological systems could also provide an overview of competence mix, vacant or understaffed shifts, and staffing resources both daily and over time. It was found to be helpful when the system could provide an overview of an employee's entire schedule, including details of when the employee had worked in other wards, the amount of individual overtime worked, shift changes, and whether employees had followed rules for scheduling.

The technological systems usually had functions to generate warnings when working time regulations were breached, such as an insufficiently long weekly rest period, a short rest in-between shifts, or too few days off during a scheduling period. While some interviewees always examined the reasons for the warnings and made necessary changes if possible, others reported that most warnings could be dismissed without any further actions. One manager stated that “*there is nothing to do about it (warnings), when they (the employees) have already switched their shifts*” (Manager 1). The reasons for the warnings were also sometimes hard to understand: “*it says that there is not enough weekly rest, and too short, too close shifts (…) maybe every week during a 10-week period, and I have to try to understand, why does it say this?*” (Staffing assistant 8).

Some technological systems were described as being sluggish and difficult to navigate. Technological errors were common, with settings and changes suddenly disappearing. Some interviewees highlighted having insufficient knowledge to use all functions. Another disadvantage was when the systems generated work schedules based on working time patterns from a period with high workload and overtime work, which resulted in unsustainable working hours that had to be adjusted manually. Also, sometimes the systems made unnecessary adjustments, resulting in a suboptimal solution for both employees and the workplace. Another problem was lack of notifications of unsustainable working hours and a poor overview for employees when planning and adjusting their schedule. When only one week at a time was visible, the employees planned too many consecutive shifts by mistake (i.e., continued planning shifts during the beginning of a week although they had worked the preceding weekend). Furthermore, the technological scheduling systems required adequate staffing to work properly.

## 4. Discussion

The results point to several factors that may be important for achieving sustainable working hours within the participatory working time scheduling process. These include the distribution and clarification of responsibilities and guidelines, leadership factors, considerations of recovery opportunities and competence mix on shifts, contradictions between employee requests and sustainable working hours, and contextual factors (e.g., staffing, work procedures, night work arrangements, and technology). The most important findings are discussed within the context of the *human/individual-technology-organisation* framework [46], which shows the complexity of scheduling in healthcare organisations where employees' *individual* preferences, *organisational* factors (e.g., demands and leadership behaviours), and *technological* solutions are interconnected.

Despite the existence of guidelines for sustainable working hours, these could be breached due to *individual* factors (e.g., employees' requests) or *organisational* factors (e.g., staffing shortage and shift vacancies). This demonstrates that sustainable working hours are not always priorities at the *individual* and *organisational* levels. The results also demonstrate that employees' requests were highly valued and sometimes prioritised over sustainable scheduling. The issue is complex. While employee influence over working hours is important in many aspects [13–20], certain scheduling characteristics are associated with poor employee sleep, health, and patient safety [26–36, 39–41]. Moreover, at the *organisational* level, employers are responsible in law for employees' health and safety at work [52], where working hours play an important role. Hence, when employees are given a high degree of responsibility for their own working hours, the resulting schedules may not be compliant with the law. Future studies are needed to examine the driving forces determining priorities in scheduling, at the levels of the *individual* (employee) and the *organisation*, and to study the consequences with respect to employee health and patient safety.

At the *organisational* level, the results identified ways in which leaders, working together with *individual* employees, could promote sustainable working hours, namely, through establishing a shared responsibility framework, fostering an individual relationship with the employee, providing support, and managing dissatisfaction. Similar to the concept of sleep leadership, that has been related to better sleep outcomes [44, 45], leadership behaviours that enable and facilitate sustainable schedules together with the *individual* (employee) might be important. Challenges for leadership were also identified, such as the difficulties of maintaining an overview of schedules when the group of employees is very large. To achieve and maintain sustainable working hours, scheduling needs to be made a priority issue for managers, with clearly defined responsibilities established within the *organisational* leadership. It was notable that few managers and staffing assistants in the current study had received formal education about healthy working hours. Organisations could benefit from the development of standardised education programs that are made a prerequisite for being responsible for working hour scheduling.

Staffing assistants, rather than managers, were most commonly involved in discussions with employees about scheduling and working hours. This sometimes placed the assistants in difficult positions. They often knew the employees' *individual* preferences and life circumstances and would try to take these into consideration, adding to the challenges of creating schedules. Assistants were often the recipients of employees' requests and complaints but had no formal responsibility for determining working hours or for decision making. Their experiences suggest a need for formal scheduling guidelines with clearer rules for sustainable scheduling, handed down to staffing assistants from higher up in the *organisation*. They also highlight the need to ensure that the staffing assistant's role, responsibility, and mandates are clearly defined.

Employees' perception (*individual* level) of unfairness in scheduling was identified as a source of dissatisfaction and as a hindrance to the scheduling process. Hence, fairness was described as important to take into consideration during scheduling. However, fairness can be a barrier to sustainable working hours, if the more sustainable scheduling solution is not the most “fair.” Therefore, *organisational* guidelines for scheduling should also specify what factors (e.g., sustainability, fairness, etc.) should have the highest priority, when staffing assistants and managers plan and adjust the schedules.

*Technological* systems were both enablers and barriers in scheduling. They often had usability issues such as unclear warnings for unsustainable scheduling that were hard to understand and easy to dismiss. However, some featured technological solutions that facilitated the scheduling process, through the automatic generation and adjustments of schedules, and by providing overviews of schedules. Technological systems have great potential to enhance sustainable scheduling and merit further development, following a user-centred systems design approach that incorporates the users' knowledge, skills, and perspectives into the design process [53]. A technological solution that considers *individual* preferences and *organisational* demands could be a useful means of support for staffing assistants' work during the adjustment process and mitigate employees' perceptions of unfairness or favouritism [25].

With regard to contextual factors, staffing shortage and short-term shift vacancies were identified as especially large barriers to the scheduling of sustainable working hours. Inadequate staffing levels (*organisational* factor) are associated with burnout and low job satisfaction among nurses, and with low patient care quality [54]. At the same time, healthcare organisations face challenges in recruiting and retaining staff [8, 9] which contributes to the staffing problems. In a previous study, including nurses from 10 different countries, satisfaction with schedule flexibility was associated with lower intention to leave the workplace [20]. Also, having flexible work hours has been cited as one reason for choosing to work for a temporary employment agency instead of working as a permanently employed nurse [55]. Hence, offering employees the possibility to participate in scheduling might increase intentions to stay in the organisation. However, it is important that the process is optimised to meet the needs of both the employees and the organisation, and that it does not result in working hours that might jeopardise employee health and patient safety. Optimisation will be supported by taking into account the interactions between individual, organisational, and technological factors highlighted in this study, thereby promoting the retention of nurses.

Sufficient staffing is an essential prerequisite for achieving sustainable working hours within a participatory scheduling system. The use of temporary agency personnel was a common solution to staffing shortages. However, there was a risk of such staff working excessive or unhealthy hours if, for example, managers and/or staffing assistants lacked a full overview of their working hours. Such cases highlight the need to pay special attention to sustainable scheduling for temporary personnel. In addition, mixing temporary and permanent nurses in work teams might trigger social comparisons and envy and affect communication within nursing teams. Organisations should seek to address such issues when using temporary agency staff by, for example, striving for transparency in how resources are allocated, promoting fairness perception, and working to promote exchange of experiences and knowledge to foster mutual learning [56].

The results also demonstrated that working hours are highly intertwined with contextual factors, such as the work procedures on the wards (*organisational* factor). For example, consistent with previous findings [57], quick returns were believed to facilitate work on the morning shift, leading some *individuals* to prefer those shift combinations. Thus, the way in which work procedures are organised can influence preferences for certain shift combinations, while the removal of certain shift combinations may hinder work procedures and diminish employees' satisfaction. Thus, a framework for complex interventions should be used when evaluating changes to working hours that also takes into account what impact the intervention has in addition to its intended outcome and considers how it interacts with the context where it is implemented [58].

A shared responsibility framework (i.e., active engagement by all persons involved in the scheduling process) was regarded as essential for the process to run smoothly. However, employees' engagement during planning and adjustment of schedules was sometimes felt to be lacking. One suggested explanation for employees' failure to engage was the absence of allocated time within the workday for scheduling activities. Increasing employee engagement in the scheduling process and strengthening the sense of shared responsibility will be challenging, but it is likely that employees' engagement is partly dependent on *organisational* factors (e.g., allocated time, degree of met requests, leadership behaviours, education, and support). Engagement can thus be considered within the context of the interaction between the *human* and the *organisation* [46], highlighting the need for organisational changes or interventions. Further research is needed to identify organisational changes that could motivate employees to take greater responsibility for formulating their own work schedules.

### 4.1. Methodological Considerations

The findings are based on a rich set of data, with information repeating itself in interviews, indicating that the number of informants was sufficient [59, 60]. One potential limitation is the use of multiple interview methods, although the quality of the interviews does not vary. Trustworthiness [61] in this study was ensured according to the following criteria. (1) Credibility (the fit between researcher's views and the representation of them) was obtained by researcher triangulation. Several researchers conducted the analysis, involving peer debriefing with external checks on the research process and examination of referential adequacy where the results were checked against the raw data conducted as the last step in the analysis. (2) Confirmability was accomplished by explaining and describing the theoretical, methodological, and analytical choices made throughout the manuscript. Moreover, the findings were demonstrably derived from the data, as shown by the provision of quotations. (3) Dependability was assured by the clear descriptions of the analysis process, enabling the reader to evaluate the process. (4) Reflexivity was addressed by involving authors from multiple disciplines in the analyses. All of the authors involved in the analyses were female, two of whom were experts in working hours and participatory scheduling and the third was an expert in the conduct of qualitative research. The fourth author (male), associate professor and an expert in working hours and participatory scheduling, was involved in the conceptualisation of the study and the preparation of the manuscript.

The authors frequently discussed their preunderstanding throughout the analysis process. Professional preunderstanding is necessary for deeper understanding of the context and the interviews, but they carry a risk that familiar facts may be overlooked. The text was read through several times, and our preunderstanding was discussed throughout the analysis process.

### 4.2. Limitations

Some limitations of this study should be noted. Firstly, the vast majority of the participants were women, reflecting the fact that healthcare is a female-dominated occupational sector in Sweden. Secondly, as only 4 out of 21 regions in Sweden were represented, key issues may have been neglected. However, the sample was drawn from regions of different sizes and locations, thus providing data from a broad range of contexts, suggesting that the results are transferable to other healthcare settings. Finally, as the data collection took part during the COVID-19 pandemic, it is possible that this has affected participants' views, although the focus of the interviews was on normal operations. Experiences referring to scheduling during the COVID-19 outbreak and peaks were excluded from the analysis.

## 5. Conclusions

Participatory working time scheduling offers potentially significant benefits for healthcare organisations that are facing major challenges in recruiting and retaining staff. However, to ensure sustainable working hours within the context of participatory scheduling, it is important to address a range of factors at multiple levels of the organisation. The factors identified in this study include clarifying responsibilities between employees, staffing assistants, and managers; making working hours a priority issue for leaders; defining clearer guidelines for sustainable scheduling (including adjustments of schedules) that are endowed from higher up in the organisation; allocating time for scheduling; and increasing engagement and involvement of the employees in the scheduling process. In addition, contextual factors need to be addressed, such as adequate staffing levels, working procedures on the wards, working hour arrangements for night work, and technological solutions. Achieving sustainable working hours within the context of participatory scheduling requires targeting multiple levels of the organisation. Future research should investigate the impact that the factors identified in this study have upon realised working hours (e.g., through the study of payroll data) and upon employee health. In addition, research is warranted that addresses participatory scheduling from the employees' perspective.

## Figures and Tables

**Table 1:** Description of the analysis process according to the six phases in thematic analysis as described by Braun and Clarke [49].

Phase 1: familiarisation with data	Data were read through by all authors separately to grasp the whole, which was a reflective phase including writing notes and own reflections
Phase 2: generating initial codes	Data were coded separately, and the authors took notes about their own thoughts
Phase 3: searching for themes	The codes were discussed by all authors and searching for themes started
Phase 4: reviewing themes	The interviews and codes were revised again by all authors, first separately and then in discussion with each other, and the themes were reviewed once again
Phase 5: defining and naming themes	The final themes were identified, and their content was described
Phase 6: producing the report	The content of the themes and subthemes was formed and was checked against the raw data one last time

**Table 2:** Overview of main themes and subthemes.

Main themes	Subthemes
Organisation of the scheduling process	Distributed responsibilities and decision making
	Time-consuming

Active leadership	Establishing a shared responsibility framework and fairness
	The individual relationship with the employee: continuous dialogue, mutual problem solving, and adaptations.
	Managing dissatisfaction
	Education, support, and clear scheduling rules

Balancing sustainable working hours, employees' scheduling requests, and competence needs	Official/unofficial guidelines for sustainable working hours
	Employees' scheduling requests versus sustainable working hours
	Considering recovery opportunities
	Competence mix on shifts

Contextual factors	Staffing levels, short-term absence, and solutions
	Working procedure at the wards
	Working time arrangements for night work
	Technological enablers and barriers for sustainable working hours

## Data Availability

The authors cannot share the raw data (interviews) since the participants have not agreed to that in the consent form.

## Supplementary Materials

Supplementary Materials The full interview guide including all questions asked to managers and staffing assistants in the interviews is provided in Appendix 1. This study adhered to the Consolidated criteria for reporting qualitative research (COREQ) [48]. The COREQ checklist is provided in Appendix 2.
