# Supplementary material for: Managing Sustainable Working Hours within Participatory Working Time Scheduling for Nurses and Assistant Nurses: A Qualitative Interview Study with Managers and Staffing Assistants
Source: J Nurs Manag. 2023 Dec 9;2023:8096034. doi: 10.1155/2023/8096034 (PMC11918713; doi:10.1155/2023/8096034)
Supplement: Supplementary Materials — The full interview guide including all questions asked to managers and staffing assistants in the interviews is provided in Appendix 1. This study adhered to the Consolidated criteria for reporting qualitative research (COREQ) [48]. The COREQ checklist is provided in Appendix 2. [file 8096034.f1.zip › Appendix 1 Interview guide.docx]

**The interview guide**

| **Main questions** | **Follow up-questions** |
| --- | --- |
| **Questions for both staffing assistants and managers** | |
| Demographics | Age, sex, profession, formal education, education in scheduling/working hours, experience (years) of working with scheduling |
| What kind of working time arrangement do you have at your workplace? | - How do employees propose their schedules? - For how long time periods do you plan schedules? - What do you think about your working time arrangement? - How is the adjustment process carried out? - How do you use vetoes? |
| Do you follow any guidelines or rules during scheduling? | - What are they? - How does it work to apply them in practice? - Can you describe any conflicts regarding these? - (If not mentioned) How do you adhere to working time regulations? - What other rules are there, not only in line with working time regulations? |
| What kind of technical support do you have? | - How does it work? - What are the pros and cons in the system? How does it help you? - What kind of support would you like to have in the system? - Can the system generate warnings for unsuitable shifts or shift combinations? Which ones? - If a warning appears, what do you do? |
| What kind of conflicts do you meet about scheduling in the workplace? | - How are the conflicts handled? - *Questions for manager only: Between whom are there conflicts (employees and/or staffing assistants)? What are the consequences of the conflicts?* |
| What support would you like in your work as a staffing assistant/manager? | - Technological? - Knowledge? - Organisational? - Other? |
| In general, how could the scheduling process be improved at your workplace? |  |
| Would you like to add something that we have not discussed? |  |
| **Specific questions for staffing assistants** | |
| Tell me about your work as a staffing assistant? | - During the adjustment process, how do you manage understaffed or overstaffed shifts? What criteria are the adjustments usually based on? - How do you manage shift vacancies appearing at short notice? - Do you sometimes correct schedules? What do you change then? - What are the challenges? - What is working out well? - Would you need more support of some kind? |
| How do you follow-up the schedules? | - Do you receive feedback about the planned schedules? - Do you follow-up schedules after they have been put in production? |
| What are the main challenges in scheduling? | - How do you prioritise between production needs, competence, competence mix, continuity of care, fatigue, recovery opportunities and safety? |
| **Specific questions for managers** | |
| How do you work with scheduling as a manager? | - How is the work distributed between the manager and the staffing assistant? - Do you sometimes not approve a schedule? When does that happen? - Do you sometimes correct schedules? What do you change then? - When are you involved in covering short-term shift vacancies? How do you do that? - How do you work with schedules with regard to recovery and safety? - Are working hours included in the systematic work environment management? |
| How do you follow-up the planned schedules? | - How big discrepancy is there between the planned schedule and the employees actual work hours? - How do you follow-up schedules based on a recovery and safety perspective? |
| Do you usually follow-up the working hours with your employees? | - How? - At the staff appraisal? - How do you discuss recovery and safety? |
| What is the biggest challenge in creating good schedules? |  |
